# Bundle-Specific Axon Diameter Index as a New Contrast to Differentiate White Matter Tracts

**DOI:** 10.3389/fnins.2021.646034

**Published:** 2021-06-15

**Authors:** Muhamed Barakovic, Gabriel Girard, Simona Schiavi, David Romascano, Maxime Descoteaux, Cristina Granziera, Derek K. Jones, Giorgio M. Innocenti, Jean-Philippe Thiran, Alessandro Daducci

**Affiliations:** ^1^Signal Processing Lab 5, École Polytechnique Fédérale de Lausanne, Lausanne, Switzerland; ^2^Cardiff University Brain Research Imaging Centre, Cardiff University, Cardiff, United Kingdom; ^3^Translational Imaging in Neurology (ThINk) Basel, Department of Biomedical Engineering, University Hospital Basel and University of Basel, Basel, Switzerland; ^4^Neurologic Clinic and Policlinic, Departments of Medicine, Clinical Research and Biomedical Engineering, University Hospital Basel and University of Basel, Basel, Switzerland; ^5^CIBM Center for BioMedical Imaging, Lausanne, Switzerland; ^6^Radiology Department, Centre Hospitalier Universitaire Vaudois and University of Lausanne, Lausanne, Switzerland; ^7^Department of Computer Science, University of Verona, Verona, Italy; ^8^Sherbrooke Connectivity Imaging Lab, Université de Sherbrooke, Sherbrooke, QC, Canada; ^9^Neuroscience and Mental Health Research Institute, Cardiff University, Cardiff, United Kingdom; ^10^Mary MacKillop Institute for Health Research, Australian Catholic University, Melbourne, VIC, Australia; ^11^Department of Neuroscience, Karolinska Institutet, Stockholm, Sweden; ^12^Brain and Mind Institute, École Polytechnique Fédérale de Lausanne, Lausanne, Switzerland

**Keywords:** human brain, white-matter axon signature, diffusion MRI, tractography, microstructure, microstructure informed tractography

## Abstract

In the central nervous system of primates, several pathways are characterized by different spectra of axon diameters. *In vivo* methods, based on diffusion-weighted magnetic resonance imaging, can provide axon diameter index estimates non-invasively. However, such methods report voxel-wise estimates, which vary from voxel-to-voxel for the same white matter bundle due to partial volume contributions from other pathways having different microstructure properties. Here, we propose a novel microstructure-informed tractography approach, COMMIT_AxSize_, to resolve axon diameter index estimates at the streamline level, thus making the estimates invariant along trajectories. Compared to previously proposed voxel-wise methods, our formulation allows the estimation of a distinct axon diameter index value for each streamline, directly, furnishing a complementary measure to the existing calculation of the mean value along the bundle. We demonstrate the favourable performance of our approach comparing our estimates with existing histologically-derived measurements performed in the corpus callosum and the posterior limb of the internal capsule. Overall, our method provides a more robust estimation of the axon diameter index of pathways by jointly estimating the microstructure properties of the tissue and the macroscopic organisation of the white matter connectivity.

## 1. Introduction

The *white matter of the central nervous system* comprises axons with different diameters (Peters et al., 1991) organized in pathways, tracts, bundles or fascicles. Diameters correlate with: (i) the size of the parent cell body (Tomasi et al., 2012); (ii) the size and density of synaptic boutons (Innocenti and Caminiti, 2017); (iii) conduction velocity (Hursh, 1937), which together with axon length determines conduction delays between brain sites; and possibly, (iv) the frequency of firing (Perge et al., 2012). Being able to quantify and characterise these different aspects may be critical to understanding sensory, motor, and cognitive functions. In particular, as the axon diameter is strictly related to conduction velocity (Ritchie, 1982; Drakesmith et al., 2019), it is associated with the flow of information between different cortical sites and is thus a critical feature when trying to understand the relationship between the structural and functional connectivity of the brain (Honey et al., 2010). Reliable estimates of axon diameter are also of utmost importance for interpreting pathological cases (DeLuca et al., 2004; Zikopoulos and Barbas, 2013; Huang et al., 2016).

First attempts to characterize the composition of neuronal pathways in the central nervous system used *histological techniques* (Aboitiz et al., 1992; Tomasi et al., 2012; Innocenti et al., 2018) and focused on samples of animal tissue. Besides being possible only *ex vivo*, these analyses require laborious measurements of axon diameters in a few slices along the course of known pathways. Per contra, *diffusion-weighted magnetic resonance imaging* (DW-MRI) is a non-invasive technology that can provide *in vivo* structural information on white matter pathways by probing the motion of water molecules and analyzing how it is influenced by the cellular structure of the tissue (Le Bihan and Breton, 1985; Moseley et al., 1990; Beaulieu and Allen, 1994). Compared to histological measurements, this technology is faster and non-invasive. Therefore, it can be applied to the living human brain, with enormous potential in terms of information that can be recovered.

On the one hand, it is possible to estimate the course of major pathways using *tractography*; for a review, see (Jeurissen et al., 2017) and references therein. These fiber-tracking methods approximate the macroscopic trajectory of axons by seeking pathways of maximum coherence of estimates of fibre orientation derived in each voxel from DW-MRI. Each reconstructed trajectory, or streamline, represents a coherent set of axons coursing together. Despite a large number of algorithms developed, none of the existing methods can provide information about the axon diameter of the individual reconstructed fiber bundles, as tractography only reconstructs their macroscopic trajectory. On the other hand, a variety of DW-MRI biophysical models have been proposed in the literature to obtain such information at the voxel level. Pioneering work in this field was done by Assaf et al. (2008), who proposed a method to estimate *axon diameter distributions* on an *ex vivo* spinal cord sample, exploiting the simple organization of the tissue with axons having a single, known orientation. Their model, AxCaliber, was later employed to study *in vivo* the axon composition of the corpus callosum in rodents (Barazany et al., 2009). A major limitation is that the DW-MRI signal must be acquired perpendicular to the axons main orientation and, hence, it requires prior knowledge on the orientation of the bundle to study. The ActiveAx technique developed by Alexander et al. (2010) removed this constraint by probing the DW-MRI signal along multiple directions and estimating orientationally-invariant features of the axons, thus not requiring any prior knowledge on their orientation. ActiveAx extended axon diameter index estimation to the whole brain but at the price of providing estimates of the *mean axon diameter* rather than the full distribution. The model was validated in monkeys and humans (Alexander et al., 2010; Dyrby et al., 2013), *in vivo* and *ex vivo*, and the estimated *trend* of the mean diameters in the corpus callosum agreed with histology.

Despite their attractiveness, current techniques for axon diameter estimation with DW-MRI suffer from several *fundamental limitations* which render them unsuitable for estimating conduction velocity and connectomics studies in the whole brain. First, the estimation is performed voxel-wise and independently in each imaging voxel, neglecting the fact that axons are continuous three-dimensional structures that are not limited to the extent of the voxel. This makes it impossible to infer the full course of the axons passing through that location or whether the estimated values correspond to distinct fiber bundles. Second, most methods implicitly assume a single axon population inside a voxel and cannot cope with complex fiber configurations such as crossing and fanning. In such voxels, [estimated to be as high as 90 % of all white-matter voxels (Jeurissen et al., 2013)], the models provide biased estimates as they suffer from severe overestimation of the axon diameters (Alexander et al., 2010), limiting de facto their applicability to specific areas of the brain, e.g., mid-sagittal plane of the corpus callosum. Recent advances extended these models to multiple fiber populations (Barazany et al., 2011; Zhang et al., 2011a; Auria et al., 2015; Farooq et al., 2016) and orientation dispersion (Zhang et al., 2011b), allowing for a more accurate estimation in complex fiber configurations. Although these methods showed consistent differences in the axon diameter index estimation from various axonal bundles, they remain limited to voxel-wise estimates, and are unable to recover bundle-specific methods. It would be desirable to obtain an accurate estimation along bundle trajectories, and in all white matter voxels, allowing for the characterization of the axon composition of individual fiber bundles. Lastly, the accuracy of the estimates crucially depends on the strength of the diffusion gradients that can be generated by the MRI scanners (Dyrby et al., 2013; Nilsson et al., 2017; Jones et al., 2018; Huang et al., 2020; Paquette et al., 2020) and other acquisition protocol (Gore et al., 2010; Siow et al., 2013; Kakkar et al., 2018; Xu et al., 2014, 2016; Drobnjak et al., 2016; Fan et al., 2020; Veraart et al., 2020), which affects the accuracy of the parameters as well (Drobnjak et al., 2010; Harkins et al., 2021); conventional human scanners are equipped with gradient systems up to 80 mT m^−1^, which do not provide the required sensitivity to axon diameters (Novikov et al., 2018; Veraart et al., 2020).

In this paper, we propose a novel method to overcome the above limitations and enable, for the first time, *a non-invasive characterization of an invariant value of axon diameter index per streamline* in the living human brain. Our method combines tractography with a microstructure model of the neuronal tissue and uses DW-MRI data acquired with a 3 T Connectom scanner capable of exploiting diffusion gradients up to 300 mT m^−1^. We demonstrate the favourable performance of our method comparing our estimates with existing histologically-derived measurements (Caminiti et al., 2009) performed in the corpus callosum and the posterior limb of the internal capsule. Estimating bundle-specific axon diameter index within each voxel of the whole white matter would represent a major advance in neuroscience, as this could shed more light on the relation between structural and functional connectivity (Honey et al., 2010) and improve our understanding of brain dynamics.

## 2. Theory and Background

### 2.1. Voxel-Wise vs. Bundle-Specific Axon Diameter Estimation

**Figure 1:**
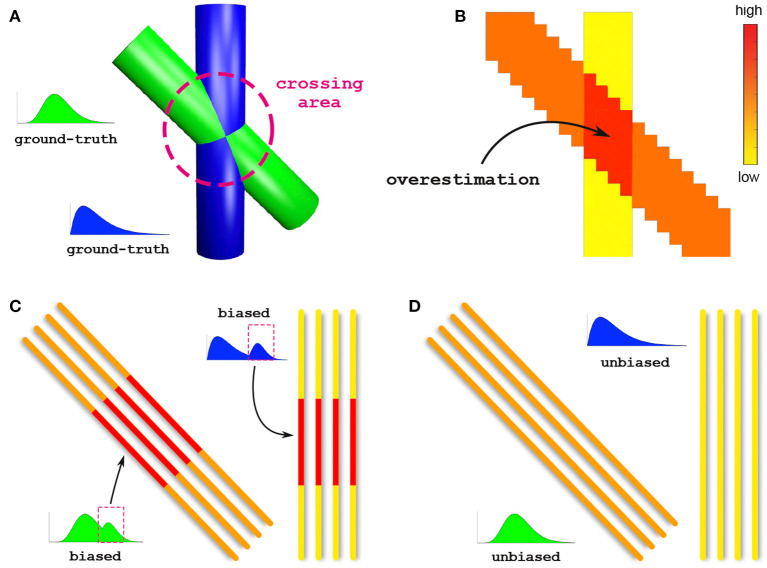
*Voxel-wise vs. bundle-specific axon diameter estimation*. **(A)** Schematic illustration of two crossing fiber populations characterized by different compositions: the green bundle contains larger axons than the blue. **(B)** Axon diameter estimation using a voxel-wise approach; for simplicity, we report the estimated mean diameters. The arrow points to the crossing region where such methods are known to especially suffer from overestimation. **(C)** Characterization of the axon diameter of a white matter bundle is typically done by averaging, along its entire course, the values previously estimated in every voxel; this indirect procedure is affected by such overestimated voxels and leads to biased results. **(D)** Estimation of bundle-specific axon diameter.

To illustrate the importance of bundle-specific axon diameter index estimation, let us consider the simple example in Figure 1. This synthetic dataset consists of two crossing fiber populations characterized by different axon compositions, with the green bundle containing larger axons than the blue one (Figure 1A). Today, the axon diameter index of a bundle is characterized using *tractometry*. This procedure indirectly approximates bundle-specific statistics by first estimating the axon diameter index with voxel-wise techniques in every voxel of the image (Figure 1B). For simplicity, we report only the estimated mean values rather than the full distributions. Then, the representative value of such a metric, for a given bundle, is obtained by *averaging these values* in all the voxels that are traversed by the streamlines belonging to the bundle. The purpose of this work is to develop a novel technique capable of estimating *bundle-specific statistics*, thus allowing us to obtain more reliable estimates of its axon composition (Figure 1D).

### 2.2. Microstructure-Informed Tractography

Even though DW-MRI is a quantitative imaging modality by nature, the sets of streamlines reconstructed by tractography are not truly quantitative (Jones and Cercignani, 2010; Jbabdi and Johansen-Berg, 2011; Jones et al., 2013). Microstructure-informed tractography (Sherbondy et al., 2009, 2010; Smith et al., 2013, 2015; Pestilli et al., 2014; Daducci et al., 2015b, 2016; Girard et al., 2017) is a recent methodological advance which aims to overcome such limitations by complementing tractography with biophysical models of the tissue microstructure. One of the recent proposed methods is the *Convex Optimization Modeling for Microstructure Informed Tractography* (COMMIT) (Daducci et al., 2015b). COMMIT assigns contibutions to the signal to each reconstructed streamline according to a microstructural forward-model and attempts to express all the acquired DW-MRI signals as a linear combination of the contributions arising from the whole set of streamlines:

(1) $$\begin{array}{r} \text{y}=\text{A}x+\eta , \end{array}$$

where ***y*** contains the DW-MRI measurements in all voxels of the white matter, **A** is a matrix that accounts for the signal contributions of the streamlines in each voxel according to a given multi-compartment model (Panagiotaki et al., 2012) (possibly in addition to local voxel-wise contributions of tissue compartments, e.g., cerebrospinal fluid) and η is the acquisition noise. The unknown contributions ***x*** of all the compartments can then be efficiently estimated by solving the inverse problem using non-negative least squares:

(2) $$\begin{array}{r} \underset{x\geq 0}{\mathrm{argmin}}||Ax-y|{|}_{2}^{2}. \end{array}$$

Similarly to other filtering approaches, COMMIT assumes that the contributions of the streamlines are constant along their trajectories. More information on the method can be found in the original COMMIT manuscript (Daducci et al., 2015b).

## 3. Materials and Methods

### 3.1. Bundle-Specific Estimation

**Figure 2:**
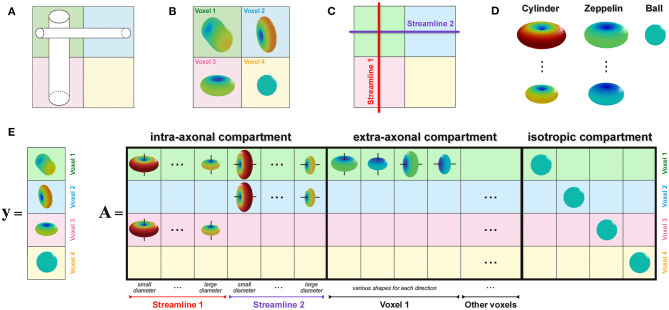
*How to enable estimation of bundle-specific axon diameter index*. **(A)** Simple crossing configuration of two fiber populations with different axon compositions, i.e., the vertical one is composed of larger axons than the horizontal, to illustrate the construction of the proposed formulation. **(B)** Corresponding DW-MRI signal in four representative voxels. **(C)** Example of two possible streamlines reconstructed with tractography. **(D)** Visual representation of the response functions in the Cylinder-Zeppelin-Ball forward model for each compartment. **(E)** The vector ***y*** contains a concatenation of the DW-MRI signal acquired in all voxels, while the matrix ***A*** is constructed by combining the response functions with the local orientations of the streamlines in each voxel.

To enable estimation of the axon diameter index of individual bundles, similarly to the recently proposed COMMIT-T_2_ method (Barakovic et al., 2021), we extended the COMMIT framework with the Cylinder-Zeppelin-Ball model (Panagiotaki et al., 2012). The new formulation, COMMIT_AxSize_, is presented in Figure 2. The proposed method *considers each streamline as consisting of a population of axons with an unknown distribution of diameters*, which must be estimated. The forward model (columns of the matrix **A**) was the DW-MRI signal arising from axons represented as parallel cylinders oriented in parallel to the tangent to the streamline in the voxel and with fixed diameters and fixed longitudinal diffusivity *d*_∥_. To account for different contributions arising from axons with distinct diameters, we considered 12 columns for each streamline corresponding to 12 cylinders with equally-spaced diameters in the range 1.5 μm to 7 μm. We modeled the extra-axonal compartment with anisotropic tensors, i.e., Zeppelins, having the same longitudinal diffusivity *d*_∥_. To capture different geometries of the extra-axonal space in every voxel, we considered multiple Zeppelins in every voxel, each with a distinct perpendicular diffusivity *d*_⊥_. Moreover, a distinct set of Zeppelins was included in **A** for every principal diffusion direction in a voxel. Finally, the cerebrospinal fluid was modeled as an isotropic tensor, i.e., Ball, with fixed diffusivity *d*_*iso*_; an independent contribution was assigned to each voxel. The physical parameters were set according to values found in the literature (Alexander et al., 2010; Zhang et al., 2011b, 2012; Le Bihan and Iima, 2015): *d*_∥_ = 1.7 × 10^−3^ mm^2^ s^−1^, *d*_*iso*_ = 3.0 × 10^−3^ mm^2^ s^−1^, and four reasonable values equally-spaced from 0.5 × 10^−3^ mm^2^ s^−1^ to 1.0 × 10^−3^ mm^2^ s^−1^ for *d*_⊥_.

The *axon diameter index of a streamline*, can be estimated from the coefficients **x** computed by COMMIT_AxSize_ as done in the ActiveAx_AMICO_ method (Daducci et al., 2015a); in fact, the 12 contributions corresponding to a given streamline represent its *volume-weighted cylinder diameter distribution*. Unlike in Assaf et al. (2008), no assumptions are made on the axon diameter distribution to be estimated. The cylinder diameter distribution can be defined for a bundle, i.e., a group of streamlines coursing through a specific region of interest (ROI). Hence, we grouped streamlines sharing the same anatomical pathways in bundles as defined by an anatomical atlas. We then calculated the axon diameter index of a bundle by performing the weighted sum, column by column, of the cylinder signature of all streamlines of the bundle.

To facilitate visual inspection of the results, we extended the Axon Diameter Index (ADI) (Alexander et al., 2010) to streamlines (sADI), which is the mean of the distribution, and colored all streamlines accordingly. To compute the sADI, we excluded the contributions of the smallest (1.5 μm) and the biggest (7 μm) cylinder diameters. This is for two reasons: i) the used DW-MRI acquisition was shown to be insensitive to diameters smaller than 2 μm (Nilsson et al., 2017). ii) We found that the smallest cylinder captures, only partially, the signal of axons from 0 μm to 1.5 μm, and the biggest cylinder captures the signal of axons above 7 μm; hence, the coefficients corresponding to those columns of ***A*** are unreliable for the computation of the sADI. Simulations were performed to validate this assumption, see Supplementary Materials.

### 3.2. Data Acquisition

#### 3.2.1. *In-vivo* Human Data

*In vivo* human data were acquired from 3 healthy volunteers on a Siemens Connectom 3 T MRI system (Cardiff University Brain Research Centre, Cardiff, Wales). The studies involving human participants were reviewed and approved by The School of Psychology Ethics Committee, Cardiff University. All participants provided written informed consent to participate in this study. Each subject was imaged five times over 2 weeks using the same DW-MRI acquisition protocol. The DW-MRI acquisition protocol used is the following: echo-time (TE) 80 ms, repetition time (TR) 3.900 ms, matrix size 110 × 110, 2 mm isotropic resolution. Other protocol parameters are reported in the Table 1.

**Table 1:** DW-MRI acquisition protocol parameters.

***b*-value (s mm^−2^)**	**δ (ms)**	**Δ (ms)**	**G (mT m^−1^)**	**directions**
1,000	7	17.3	138	30
4,000	7	17.3	276	60
1,000	7	30	102	30
4,000	7	30	203	60
1,000	7	42	85	30
4,000	7	42	169	60
1,000	7	55	74	30
4,000	7	55	175	60

*The images were acquired using a 2 mm isotropic resolution and a matrix size of 110 × 110. The echo-time (TE) was 80 ms and the repetition time (TR) was 3900 ms*.

Five non-diffusion weighted images (b0) were acquired, including one in reverse phase encoding. A 1 mm isotropic resolution T1-weighted anatomical image was also acquired, using a magnetization-prepared rapid acquisition gradient echo (MPRAGE) sequence: TE = 2 ms, inversion time = 857 ms, TR = 2.300 ms, matrix size = 256 × 256, flip angle = 9°.

#### 3.2.2. Simulation Data

A numerical phantom was generated with a 45° crossing configuration between two bundles, from which, main directions were obtained at each voxel. The intra-axonal and extra-axonal signals were generated separately and then merged to generate unique numerical phantom (Rensonnet et al., 2018). For each bundle, the DW-MRI intra-axonal signal was simulated, using a distribution of parallel cylinders (Van Gelderen et al., 1994) following a gamma distribution. The first bundle had a gamma distribution with volume weighted mean diameter of 2.70 (shape = 3.2734 and scale = 0.2556). For the second bundle, the volume weighted mean diameter was 4.00 (shape = 3.5027 and scale = 0.3655). The extra-axonal signal was generated using a tensor with perpendicular diffusivity adapted to the local intra-axonal volume fraction, following the tortuosity approximation (Szafer et al., 1995). In single fiber voxels, the intra-axonal signal fractions were set to 0.3 and 0.6 for the vertical and diagonal bundles, respectively (i.e., the extra-axonal signal was a tensor with perpendicular diffusivity equal to 0.7 × *D* and 0.4 × *D*, respectively). The crossing voxels had an intra-axonal volume fraction of 0.9 (i.e., the extra-axonal signals were generated with a perpendicular diffusivity equal to 0.1 × *D*). All signals were summed to have a total signal fraction of 1 in each voxel. The diffusivity of the simulations were fixed to *D* = 1.7 × 10^−3^ mm^2^ s^−1^ (Alexander et al., 2010; Zhang et al., 2011b, 2012), both for intra-axonal and extra-axonal signals. The resulting dataset was corrupted with various levels of Rician noise. Furthermore, four additional dataset were generated, adding voxel-wise dispersion using a Watson distribution with *k* = 4, 8, 12, 16 (Zhang et al., 2011b, 2012).

For the voxel-wise estimation, the ADI for each voxel was estimated with the ActiveAx method (Alexander et al., 2010) implemented in the AMICO framework (Daducci et al., 2015b). For the COMMIT_AxSize_ method, the bundle-specific axon diameter index were estimated using both the ground-truth bundle trajectories and using the MrTrix3 second-order integration over Fiber Orientation Distribution (iFOD2) algorithm generating approximately 1,000 streamlines per bundle. Streamlines not ending at the bundle extremities were removed before processing with COMMIT_AxSize_.

### 3.3. Data Pre-processing

The anatomical T1-weighted image was registered to the preprocessed average b0 image using FSL/FLIRT (Jenkinson and Smith, 2001) using rigid-body registration. The white matter and gray matter masks were estimated using FSL/FAST (Zhang et al., 2001). The brain cortical parcellation was performed using FreeSurfer (Destrieux et al., 2010). The DW-MRI images were corrected for magnetic field inhomogeneities, eddy currents (Andersson and Sotiropoulos, 2016) and motion using the TOPUP (Graham et al., 2017), and EDDY tools of FSL (Jenkinson and Smith, 2001). Subsequently, gradient non-linearity correction was performed (Jovicich et al., 2006; Rudrapatna et al., 2021). The shell with diffusion time Δ = 17.3 ms, *G* = 276 mT m^−1^ and b-value = 4000 s mm^−2^ was used to perform Constrained Spherical Deconvolution (CSD) (Tournier et al., 2007). Tractography was then performed using iFOD2 algorithm (Tournier et al., 2012), generating 10,000,000 streamlines seeding from the white matter mask. Streamlines not reaching the gray matter mask were removed. To make the computational time practical, a sub-set of 300,000 streamlines was randomly selected for each DW-MRI dataset.

### 3.4. Analysis of Specific Neuronal Connections

**Figure 3:**
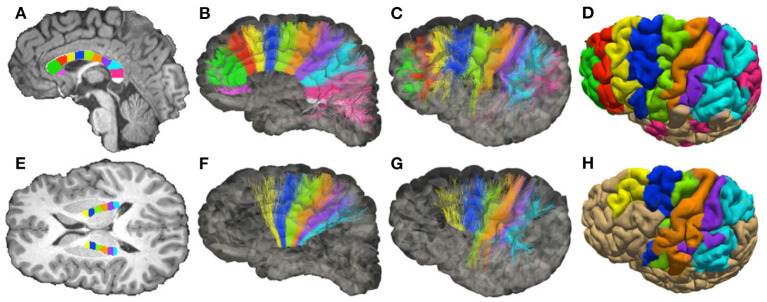
Topology of fibers in the Corpus Callosum (CC) and posterior limb of the internal capsule (PIC), reconstructed with DW-MRI tractography of a single healthy volunteer. **(A)** Subdivision of the mid-sagittal section of the CC in 11 sectors (corresponding to ROIs), see Supplementary Figure 9. **(B,C)** Streamlines colored according to the corresponding ROIs (medial and lateral views of the hemisphere). **(D)** Projection of the streamlines onto the pial surface. **(E)** Subdivision of PIC in 6 sectors (ROIs). **(F,G)** Streamlines colored according to the corresponding ROIs (medial and lateral view of the hemisphere). **(H)** Projection of the streamlines onto the pial surface.

We report the sADI estimated for the streamlines passing through individual sectors of corpus callosum (CC) and of the posterior limb of the internal capsule (PIC). The midsagittal section of the CC was outlined using to the FreeSurfer parcellation, and the transverse section of the PIC was manually outlined on the T1-weighted image by an expert anatomist. The skeletons (longitudinal centerline) of both regions were computed and then subdivided into equally-spaced segments. The boundaries of each sector were drawn roughly perpendicular to the skeleton by associating all voxels within the outlines to their closest segment. We fixed 11 regions of interest (ROIs) in the CC showed in Figure 3A, and 6 ROIs in the PIC showed for each hemisphere in Figure 3E.

Our *in vivo* study is focused on two well-characterized axonal tracts: the CC and the PIC. The CC has been well studied in the past with different methodologies, including DW-MRI (Barazany et al., 2009; Alexander et al., 2010). The PIC has been less studied with DW-MRI but is extremely important since it is traversed by cortico-descending axons involved in motor control, whose lesions lead to irreversible paralysis. Moreover, we concentrate the analysis on these two bundles since they are known to have a sufficiently large axon diameter, **Figure 6**. To study the topology of bundles, the CC and the PIC were segmented and subdivided in, respectively, 11 and 6 equal ROIs normalized for different individuals as described in section 3.4. The streamlines passing through regions of interest (ROIs) corresponding to these sectors were selected, and we analyzed their projections to and from the cortex. These projections correspond to corticofugal and corticopetal (for the CC) connections since DW-MRI does not distinguish the direction of the connections. Bundles of streamlines systematically organized from anterior to posterior connect the CC to similarly ordered slabs of cortex extending from the cingulate gyrus to the lateral sulcus (see Figure 3). This is usually neglected the aspect of CC topology, albeit already shown by tracer injections in the CC of the cat (Nakamura and Kanaseki, 1989), and is compatible with the ordering of CC connections already described with DW-MRI (Hofer et al., 2015). Also, anteroposteriorly organized bundles of streamlines connect the sectors of PIC to anteroposterior cortical territories, compatible with the topology shown by tracer injections and DW-MRI in monkeys (Morecraft et al., 2017) and DW-MRI in humans (Archer et al., 2018).

### 3.5. Comparison With Histology

The fiber composition of the CC obtained with COMMIT_AxSize_ was compared with postmortem measurements from a previous study (Caminiti et al., 2009); however, to evaluate the impact of histological sampling one of the sectors was measured again (see Supplementary Figure 8). Between 451 and 1934 axons stained for myelin were measured in CC sectors crossed by axons connecting the prefrontal, motor, parietal and visual cortices. From the histological data, we estimated the histogram of diameters in each sector. However, since DW-MRI estimates the signal fractions that are related to the volume occupied by axons of different diameter, not their number, the data was converted to volume-weighted distributions, to allow comparison with the DW-MRI estimates. In the absence of human data, the *in vivo* estimates of the PIC were compared with measurements of axons stained for myelin in the monkey PIC (Innocenti et al., 2018).

## 4. Results and Discussion

### 4.1. Numerical Simulations

**Figure 4:**
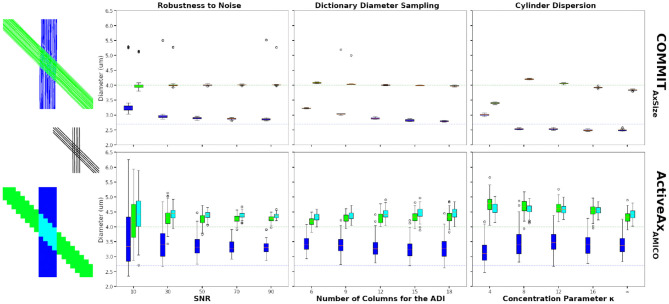
Axon diameter indexes (ADI) estimated on the 45-degrees crossing synthetic phantom. The figures show the bundle-specific ADI estimated using the COMMIT_AxSize_ method (top row) and the voxel-wise ADI estimated using the ActiveAx_AMICO_ method (bottom row). The boxplots show the results 50 different Rician noise realisations for COMMIT_AxSize_ and 100 voxels sampled in each single bundle areas and in the crossing area, for the ActiveAx_AMICO_ method. The mean ground truth cylinder diameter of the green and blue bundles are 4.0 μ*m*, and 2.7 μ*m*, respectively (colored dashed line). The first boxplot column show the estimated ADI at various SNR, the second column using various number of columns in matrix A to compute the ADI and the last column shows the estimates for data with various levels of dispersion.

Figure 4 compares the estimated ADI obtained using the conventional voxel-wise procedure and the proposed bundle-specific COMMIT_AxSize_ on the numerical phantom described in Section 3.2. The results from COMMIT_AxSize_ show more consistent estimates of the bundles mean cylinder diameter, compared to the voxel-wise method, in particular at low SNR (Figure 4 first column). Moreover, at SNR = 50 COMMIT_AxSize_ estimated on average a sADI of 2.90 μ*m* and 4.01 μ*m* compare to 3.37 μ*m* and 4.25 μ*m* for the ActiveAx_AMICO_ (the mean ground-truth diameter of each bundle is 2.7 μ*m* and 4.0 μ*m*, respectively). The estimates provided by COMMIT_AxSize_ are both more robust to noise, and closer to the real values, when compared to voxel-wise estimates.

The second columns of Figure 4 show the ADI estimates at SNR = 50, changing the dictionary diameter sampling of both methods. Results improve for COMMIT_AxSize_ increasing the sampling density, in particular for the bundle with the smallest mean diameter. Results of the voxel-wise method are similar using all dictionaries, with the best performances using 12 or 15 values. In all cases, COMMIT_AxSize_ outperformed the voxel-wise method. Although more elements in the dictionary improve the estimation, the optimisation problem becomes harder and increases the computation requirements. Nonetheless, a dictionary sampling of 12 columns in matrix A provides a reasonable estimate on synthetic data, while keeping the computation requirement feasible for *in vivo* data.

Finally, Figure 4 (third column) show the ADI estimated in the same phantom, but including various levels of dispersion (SNR = 50, dictionary diameter sampling of 12). Rather than using the ground-truth cylinder trajectories, we used probabilistic tractography to estimate their trajectories, capturing the dispersion information from the data. The rightmost boxplot shows the estimates using the probabilistic tractography with no dispersion (κ = inf). Using the probabilistic tractography streamlines, COMMIT_AxSize_ shows an underestimation of the mean diameter when compared to the ground-truth bundle trajectories. However, the increase in dispersion (lower κ value) show a systematic over-estimation of the mean diameter of the largest bundles, and little effect on the bundle with the smallest cylinder diameter. However, the trend changes at κ = 4, where both bundle ADI are estimated between 2.9 μ*m* and 3.5 μ*m*. This could be explained by the inability of probabilistic tractography to properly capture this high level of dispersion. Although COMMIT_AxSize_ cannot fully model the dispersion, the estimate along the streamlines provides more robust estimates of the bundle diameter than the voxel-wise method.

Moreover, contrary to the voxel-wise method, COMMIT_AxSize_ can disentangle bundles in crossing configurations and provide a reliable bundle-specific ADI in those areas. Something not achievable robustly with a voxel-wise method assuming a single fiber population. These numerical experiments showed the benefit of COMMIT_AxSize_ when estimating axon diameter indexes.

### 4.2. *In vivo* Data

**Figure 5:**
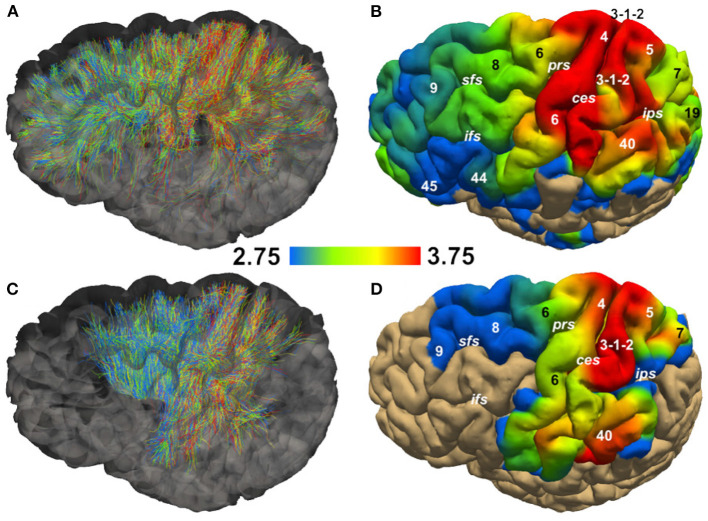
Streamline Axon Diameter Index (sADI) in the Corpus Callosum (CC) and posterior limb of the internal capsule (PIC) of a single healthy volunteer. **(A,C)** show streamlines colored according to their sADI. **(B,D)** show the projection of streamlines'sADI onto the pial surface; colors correspond to the sADI averaged across streamlines. Abbreviations: ces, central sulcus; ifs, inferior frontal sulcus; ips, interparietal sulcus; prs, precentral sulcus; sfs, superior frontal sulcus. Numbers correspond to Brodman areas.

**Figure 6:**
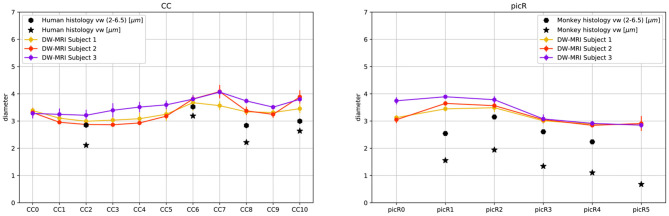
DW-MRI cylinder diameter distribution of CC and PIC sectors compared to histological mean volume-weighted axon diameter from human (CC) and monkey (PIC) histology. Star markers represent the means of volume weighted histological distributions; hexagon markers represent the means of volume weighted cylinder distributions in the range 2.0μm to 6.5μm.

Figure 5 shows the streamlines passing through the CC (A) and the PIC (C), colored following their corresponding sADI. Figures 5B,D show sADI projected onto the pial surface. In both bundles we studied, the largest sADI were found in sectors of PIC traversed by axons connecting the motor cortex (BA 4) while smaller sADI were found for other areas. This visualization reveals that streamlines with larger sADI connect the CC to the precentral gyrus, corresponding to the primary motor cortex (M1; Broadman area BA 4), the more lateral part of premotor cortex (BA 6), and the postcentral gyrus (BA 3,1,2) corresponding to the primary somatosensory cortex (S1). Streamlines with progressively smaller sADI terminate in the medial premotor cortex (BA 6) and the parietal cortex (BA 5,7 and 40) and still smaller sADI in the rostral prefrontal cortex (BA 8 and 9) and BA 44 and 45. In case of the CC, human postmortem material was used to validate the estimates obtained with our novel technique. The comparison was performed in four different ROIs. Figure 6 shows that the bundle sADI estimated with COMMIT_AxSize_ closely corresponds to the histological estimates within the DW-MRI range of sensitivity.

In monkey species (Caminiti et al., 2009; Tomasi et al., 2012), a hierarchy of axon diameters exists with thicker and faster-conducting axons connecting the motor and somatosensory cortices, thinner and slower axons elsewhere. The streamlines coursing in the PIC were color-coded as above according to their estimated sADI. These with the high sADI mapped onto the dorsal part of the precentral BA 4 (M1) and postcentral (BA 3,1,2; S1) gyrus. Progressively smaller sADI mapped onto the parietal cortex (BA 5 and 7) and the premotor cortex (BA 6) and still smaller sADI onto the rostral prefrontal cortex (BA 8 and 9). This arrangement is similar to that demonstrated with injections of anterogradely transported tracers in corresponding areas of the monkey, although in the monkey the diameter of axons originating in the precentral gyrus exceeds that of axons originating in the postcentral gyrus (Innocenti et al., 2018). Identical findings were reproduced for different sectors of the CC and PIC in three subjects and five times for each subject, Figure 6 and Supplementary Figure 10.

Current technologies restrict the resolution of axon diameters to about 2.0 μm (Nilsson et al., 2017). Nevertheless, axons with larger diameter show a detectable contrast according to our simulations, see Supplementary Materials. Despite this limitation, since large axons are found preferentially in specific pathways, their absence in the expected pathways, or abnormal presence in unexpected pathways can disclose the neural basis of specific neurological or psychiatric pathologies (DeLuca et al., 2004; Zikopoulos and Barbas, 2013; Huang et al., 2016; Judson et al., 2017; Golden et al., 2020) and, possibly, of individual skills (de Manzano and Ullén, 2018).

### 4.3. Strengths and Limitations

Our proposed bundle-specific approach allows investigation of the intrinsic axon composition of white matter pathways as opposed to sampling their composition at discrete locations along their course, making no assumptions on the axon composition of a bundle. As the DW-MRI signal in each voxel is expressed as the combined contributions of multiple intersecting streamlines, our method naturally handles the presence of different pathways within a voxel and allows their individual contributions to be decoupled. This contrasts with methods in which a ‘powder average' of the diffusion-weighted signal is taken as part of the axon diameter estimation process (Veraart et al., 2018). On the contrary, in voxels with such complex fiber configurations, the current voxel-wise estimation approaches provide biased estimates. Another advantage of our approach is that the cylinder diameter distribution of a bundle could be mapped onto the cortex where it originates (and/or terminates), eliminating the ambiguities of following axon diameters at selected locations along the white matter pathways (Assaf et al., 2008; Barazany et al., 2009; Alexander et al., 2010).

One could argue that it has been demonstrated that although the axonal diameter of single axons can undergo local changes along its trajectory (Lee et al., 2019); the axon diameter distribution of diameters in a pathway remains stable over long distances (Tomasi et al., 2012).

Nevertheless, we stress that a streamline represents a group of axons that share a similar trajectory; thus, our method estimates an average diameter for the represented group. Moreover, by discretizing the intra-axonal signal in the contributions arising from multiple impermeable cylinders (Van Gelderen et al., 1994), each streamline can be composed of a different amount of cylinders with different diameters without imposing any prior on the eventual distribution.

By decomposing the signal of each voxel into three components (intra-axonal, extra-axonal and isotropic compartments) and regularizing the intra-axonal signal fractions along streamlines, we were able to detect the signal fractions corresponding to each component. In particular, we discretized the signal coming from each cylinder diameter using the formula for impermeable cylinders of Van Gelderen et al. (1994) and what we estimated through the COMMIT_AxSize_ method was the weighting factor in front of each diameter *d*_*i*_, which is of the order of $d_{i}^{2}$ (Burcaw et al., 2015). Similarly, we discretized the signal coming from the extra-axonal compartment in two main components, parallel and perpendicular, along each principal direction. Both components were fixed using physically plausible constant values for the diffusion coefficient (Alexander et al., 2010; Zhang et al., 2012). Moreover, for the perpendicular direction, we accounted for four possible diffusion coefficients (i.e., in each voxel for each main direction we estimated five possible fractions of extra-axonal signal: one parallel to the fiber population and four perpendiculars to it). The remaining fraction of the signal in each voxel was then captured by the signal contribution of an isotropic compartment with fixed diffusivity. With these parameters, we do not account for the eventual residual time dependence of the extra-axonal diffusion tensor. However, by allowing a different fraction for each discretized value in the perpendicular direction, we account for a positive contribution of this compartment. Indeed, although we acquired data with strong gradients, we are not in a regime for which the extra-axonal signal may not be completely suppressed (Veraart et al., 2019). The model we used in the signal discretization, as well as the acquisition parameters chosen for the DW-MRI sequence, can be improved to be more sensitive to microstructure features.

Recent studies have suggested that axons may vary in diameter along their length, may present undulations and microscopic orientation dispersion (Brabec et al., 2020; Lee et al., 2020a; Rafael-Patino et al., 2020) which impacts the estimate obtained with DW-MRI (Lee et al., 2020b). The impact of those tissue properties on COMMIT_AxSize_ will be addressed in future studies. Furthermore, the volume fractions selected for our simulation experiment were limited by the choice of substrate simulator (Rafael-Patino et al., 2020). Future numerical simulations will address more complex configurations, and more realistic substrates will be constructed (e.g., varying volume fractions and diameter distributions).

In this study, we proposed to estimate the bundle-specific axon diameter index implementing the Cylinder-Zeppelin-Ball forward model. We believe that the selection of the optimal microstructure forward model and dictionary parameters could be improved in future studies. In particular, the dictionary discretization used (i.e., 12 values for the intra-axonal component) and fixed values for diffusivities, which may cause loss of accuracy (Jelescu et al., 2016), will be two aspects to explore extensively. Moreover, various tractography algorithms will be tested to build the COMMIT_AxSize_ dictionary (e.g., deterministic and probabilistic, and tractography parameters). Future works will address these aspects, including the exploiting different diffusion acquisition protocols, using similar approaches as in Drobnjak et al. (2016) and Nilsson et al. (2017), to find an optimal set of parameters and protocols improving the sentitivity to the tissue properties (Lampinen et al., 2017). Another important aspect to mention is that the COMMIT_AxSize_ may be inaccurate in the diseased brain, affected by focal lesions along white matter tracts. However, it may be applicable to developmental disorders, psychiatric disorders and neurological diseases such as epilepsy.

## 5. Conclusion

In this paper, we focused on the non-invasive characterization of the composition of central nervous system pathways in the living human brain from DW-MRI acquisitions. In particular, we tackled some fundamental limitations of current voxel-wise techniques and proposed a novel formulation to estimate the axon diameter index of a fiber bundle all along its trajectory, rather than sampling it at a few selective locations along its course. We compared our bundle-specific approach to the state-of-the-art voxel-wise methods, both on synthetic and *in vivo* human brain data, comparing our findings with histological measurements in two well-studied cortical pathways. Our results demonstrated the feasibility and the benefits of our proposed formulation. Moreover, the bundle composition estimated agree with histology and known anatomy. Further studies could extend the present approach to other pathways in the central nervous system, enhancing the human connectome enterprise (Craddock et al., 2013; Jbabdi et al., 2015; Glasser et al., 2016).

## Data Availability Statement

The raw data supporting the conclusions of this article will be made available by the authors, without undue reservation.

## Ethics Statement

Ethical review and approval was not required for the study on human participants in accordance with the local legislation and institutional requirements. The patients/participants provided their written informed consent to participate in this study. Written informed consent was obtained from the individual(s) for the publication of any potentially identifiable images or data included in this article.

## Author Contributions

MB, GG, DR, SS, MD, CG, DJ, GI, J-PT, and AD conceptualized the problem. MB and AD developed, implemented, and tested the technical framework. DJ provided the MRI data. MB and GI validated the framework. GI contextualized the framework with a biological and application-oriented perspective. MB, GG, SS, GI, and AD wrote the manuscript.

## Conflict of Interest

The authors declare that the research was conducted in the absence of any commercial or financial relationships that could be construed as a potential conflict of interest.
